# Inhibitory Effect of Allyl Isothiocyanate on Cariogenicity of *Streptococcus mutans*

**DOI:** 10.3390/ijms26157443

**Published:** 2025-08-01

**Authors:** Tatsuya Akitomo, Ami Kaneki, Masashi Ogawa, Yuya Ito, Shuma Hamaguchi, Shunya Ikeda, Mariko Kametani, Momoko Usuda, Satoru Kusaka, Masakazu Hamada, Chieko Mitsuhata, Katsuyuki Kozai, Ryota Nomura

**Affiliations:** 1 Department of Pediatric Dentistry, Graduate School of Biomedical and Health Sciences, Hiroshima University, Hiroshima 734-8553, Japan; kaneki@hiroshima-u.ac.jp (A.K.); caries0@hiroshima-u.ac.jp (M.O.); yuuya@hiroshima-u.ac.jp (Y.I.); syuumai@hiroshima-u.ac.jp (S.H.); shunyaikeda@hiroshima-u.ac.jp (S.I.); mrysk25@hiroshima-u.ac.jp (M.K.); myoshii0108@icloud.com (M.U.); chiekom@hiroshima-u.ac.jp (C.M.); kozai@hiroshima-u.ac.jp (K.K.); rnomura@hiroshima-u.ac.jp (R.N.); 2 Department of Pediatric Dentistry, Hiroshima University Hospital, Hiroshima 734-8551, Japan; higechi@hiroshima-u.ac.jp; 3 Department of Oral & Maxillofacial Oncology and Surgery, Graduate School of Dentistry, The University of Osaka, Suita 565-0871, Japan; hamada.masakazu.dent@osaka-u.ac.jp

**Keywords:** allyl isothiocyanate, bacterial growth, bacterial survival, RNA sequence, *Streptococcus mutans*

## Abstract

Allyl isothiocyanate (AITC) is a naturally occurring, pungent compound abundant in cruciferous vegetables and functions as a repellent for various organisms. The antibacterial effect of AITC against various bacteria has been reported, but there are no reports on the effect on *Streptococcus mutans*, a major bacterium contributing to dental caries. In this study, we investigated the inhibitory effect and mechanism of AITC on the survival and growth of *S. mutans*. AITC showed an antibacterial effect in a time- and concentration-dependent manner. In addition, bacterial growth was delayed in the presence of AITC, and there were almost no bacteria in the presence of 0.1% AITC. In a biofilm assay, the amount of biofilm formation with 0.1% AITC was significantly decreased compared to the control. RNA sequencing analysis showed that the expression of 39 genes (27 up-regulation and 12 down-regulation) and 38 genes (24 up-regulation and 14 down-regulation) of *S. mutans* was changed during the survival and the growth, respectively, in the presence of AITC compared with the absence of AITC. Protein–protein interaction analysis revealed that AITC mainly interacted with genes of unknown function in *S. mutans*. These results suggest that AITC may inhibit cariogenicity of *S. mutans* through a novel mechanism.

## 1. Introduction

Dental caries is one of the most prevalent and costly biofilm-associated infectious diseases affecting most of the world’s population [1]. There are improved trends in the number of dental caries infections in developed countries, but eradicating dental caries remains difficult. In addition, dental caries remains prevalent and is increasing in some developing countries undergoing nutrition transitions [2]. Many commensal bacteria that can adapt to acidic environments have been implicated in developing dental caries [3]. *Streptococcus mutans* remains the major cariogenic pathogen, with its high acid tolerance and ability to form biofilms [1]. In addition, *S. mutans* and other oral streptococci can enter the bloodstream after dental extractions, brushing teeth, and chewing, and can also lead to bacteremia or infective endocarditis [4]. Some studies have reported that *S. mutans* can be isolated from the blood samples of patients with bacteremia and infective endocarditis, which reveals that *S. mutans* is associated with systemic diseases, mainly cardiovascular diseases [4,5,6,7].

Dental expenditures cost Americans USD 55 billion out of pocket in 2018, which constitutes over 25% of all out-of-pocket healthcare expenses [8]. Dental caries represents a global socio-economic burden, and the development and discovery of materials that can be used to prevent dental caries remain pertinent to reducing these healthcare expenses. Naturally derived materials, mainly from plants, have been widely used in the pharmaceutical field because they are familiar to humans and often have a high level of safety [9]. Recent studies have reported the effectiveness of naturally derived materials for preventing dental caries worldwide [10,11,12]. Extracts derived from plants have been demonstrated to have inhibitory effects against *S. mutans* [13,14,15], positioning them as promising agents for preventing dental caries.

Allyl isothiocyanate (AITC) is an organosulfur compound abundant in cruciferous vegetables of the Brassicaceae family, including cabbage, broccoli, and mustard [16]. AITC prevents plant infections and is beneficial in food preservation due to its antibacterial properties [17]. In humans, AITC is well known to have anticancer and anti-inflammatory effects [16,18,19]. AITC also has antibacterial and antifungal properties [16,20,21] and has antibacterial effects against major oral bacteria such as *Porphyromonas gingivalis* [22]. Still, no reports have investigated its inhibitory effect against *S. mutans*. We have previously demonstrated the inhibitory effects of various naturally derived materials against *S. mutans*, and based on this experience, we verified the effect of AITC against *S. mutans* [11,12].

The aim of this study was to investigate the inhibitory effect of AITC on the cariogenicity of *S. mutans*. In this study, we focused on the effect of AITC on the antibacterial activity, bacterial growth, and biofilm formation of *S. mutans*. Additionally, we analyzed the effects of AITC on gene expression and predicted the protein interactions of *S. mutans*. On the other hand, its limitation is that it has been conducted entirely in the laboratory, and further research is needed before clinical application.

## 2. Results

### 2.1. AITC Became Extinct S. mutans in a Concentration- and Time-Dependent Manner

**Figure 1:**
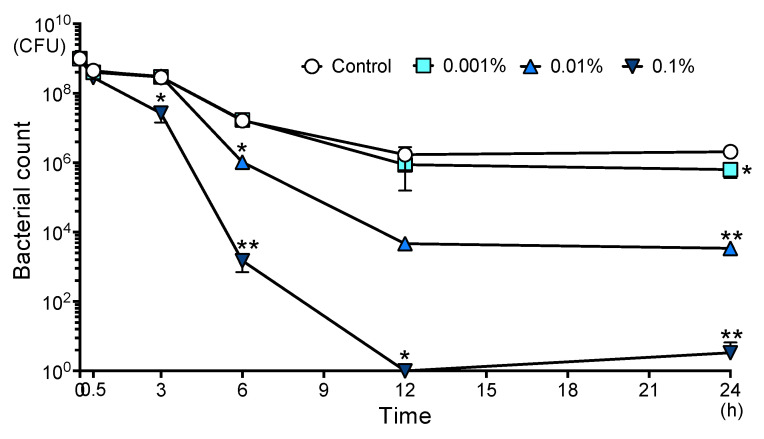
Bacterial survival of AITC on *S. mutans*. * *p* < 0.05 and ** *p* < 0.01. Bars indicate mean and SE.

Before conducting the experiment, we investigated the change in pH due to the addition of AITC and confirmed that they were both 7.5 in phosphate-buffered saline (PBS) and 0.1% AITC. The bacterial survival assay was performed using PBS as the solvent, which does not allow bacterial growth, at an initial bacterial concentration of 1 × 10^9^ CFU/mL. The results of the bacterial survival assay are shown in Figure 1. At 30 min, there was no difference in bacterial count with or without AITC. The bacterial count in 0.1% AITC was significantly lower than that in 0% AITC (control group) at 3 h (*p* < 0.05). After 6 h, the 0.01% and 0.1% AITC significantly reduced the number of *S. mutans* compared with the control group (*p* < 0.05). In addition, the number of *S. mutans* in all concentrations of AITC was significantly decreased compared with the control at 24 h (*p* < 0.05).

### 2.2. AITC Inhibited the Bacterial Growth

**Figure 2:**
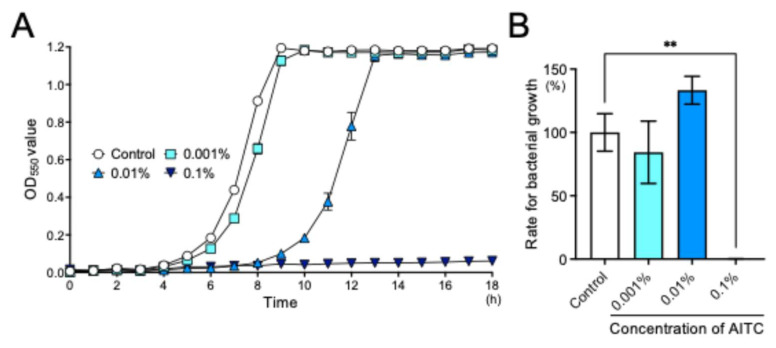
Growth inhibition of AITC on *S. mutans*. (**A**) Time course of the growth on *S. mutans*. Bars indicate mean and SE. (**B**) Rate of growth of *S. mutans* in the bacterial growth assay at 24 h, ** *p* < 0.01. The number of *S. mutans* in the control group was defined as 100%. Bars indicate mean and SE.

The bacterial growth assay was performed using Brain Heart Infusion (BHI) as a solvent, which allows bacterial growth, with an initial bacterial concentration of 1.0 × 10^7^ CFU/mL (at this concentration, no difference was observed in the OD_550_ values compared to the control group). In BHI broth, the pH of the solution did not change with or without AITC (BHI: 7.1; 0.1% AITC: 7.1). The change in the OD_550_ value of bacterial suspensions is shown in Figure 2A. The turbidity in the control group gradually increased after 4 h and reached a plateau at 9 h. In 0.001% AITC, a significant delay in bacterial growth was observed from 5 to 9 h (*p* < 0.05), and in 0.01% AITC, a statistically significant difference was observed from 4 to 12 h compared with the control (*p* < 0.01). In 0.1% AITC, almost no growth of *S. mutans* was observed for an incubation period of 18 h, and even when comparing the bacterial counts after 24 h, there was a significant inhibition of *S. mutans* growth compared to the control group (*p* < 0.01) (Figure 2B).

### 2.3. AITC Inhibited the Biofilm Formation

**Figure 3:**
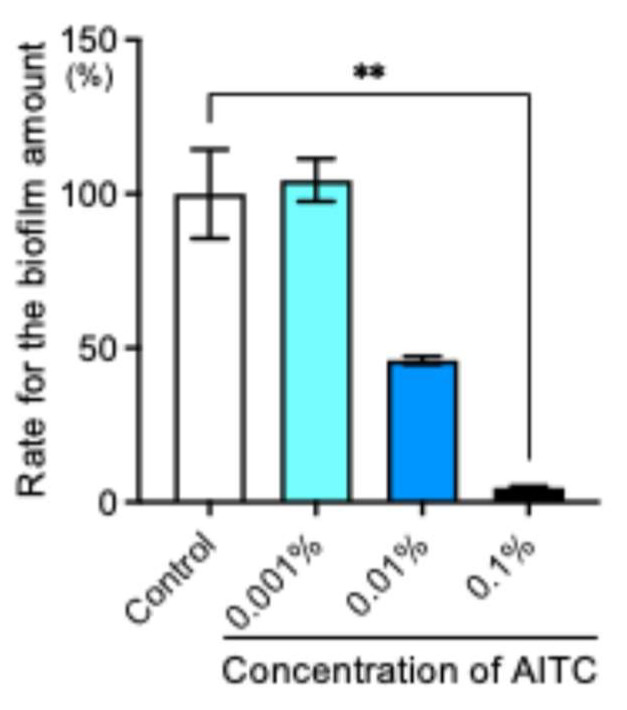
The amount of biofilm formation, ** *p* < 0.01. The amount of biofilm in the control group was defined as 100%. Bars indicate mean and SE.

The biofilm assay measured the amount of biofilm formed after 24 h of incubation in BHI containing 1% sucrose. As a result, 0.001% AITC was not different from the control group (Figure 3). On the other hand, the amount of biofilm formed significantly decreased at 0.1% (*p* < 0.01).

### 2.4. RNA Sequencing Analysis Revealed That Expression of 39 Genes Is Altered in Survival Assay and 38 Genes in Growth Assay in Three Conditions

**Figure 4:**
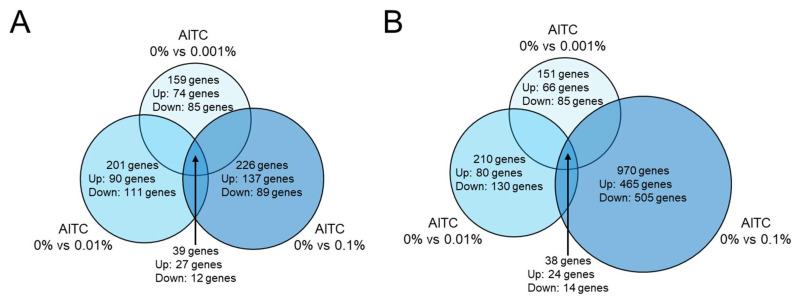
Schema of analyses using RNA sequencing of *S. mutans* treated with AITC. *S. mutans* genes whose expression levels were up-regulated or down-regulated more than 2-fold in the presence of each concentration of AITC, compared to the absence of AITC, were extracted. (**A**) Bacterial survival. (**B**) Bacterial growth.

The bacterial survival and growth assays of *S. mutans* described above were performed in the presence of each concentration of AITC (0%, 0.001%, 0.01%, and 0.1%). Then, RNA sequencing analysis was performed to comprehensively analyze the gene expression changes that AITC causes in *S. mutans*. We evaluated three conditions with several different concentrations of AITC: 0% vs. 0.001%, 0% vs. 0.01%, and 0% vs. 0.1% (Figure 4A,B). In each condition, genes up-regulated or down-regulated more than 2-fold were extracted. In bacterial survival assays, the AITC at each concentration resulted in changes in the expression of 150–250 genes. In growth assays, 0.001% and 0.01% AITC showed changes in the expression of a similar number of genes as in the survival assays, but 0.1% AITC changed the expression of approximately 1000 genes. There were 39 genes in the survival assay (27 up-regulated and 12 down-regulated) and 38 genes in the growth assay (24 up-regulated and 14 down-regulated) that were common to all three conditions (Table 1, Table 2, Table 3 and Table 4). Among these genes, *comYD*, which is involved in the regulation of *S. mutans* competence, was up regulated in the survival assay, but the functions of most of the genes were unknown.

### 2.5. Protein–Protein Interaction Network with Genes Whose Expression Was Altered by AITC Was Constructed

**Figure 5:**
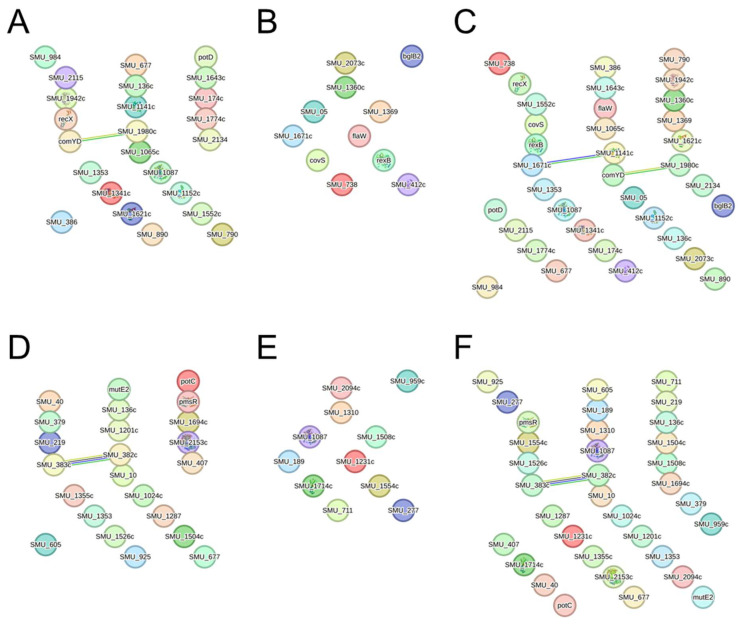
Protein–protein interaction (PPI) network analysis using genes whose expression was altered in *S. mutans* in the presence of all concentrations of AITC compared to the absence of AITC. In the bacterial survival assay, 27 genes whose expression levels were up-regulated by more than two-fold (**A**), 12 genes whose expression levels were down-regulated by more than two-fold (**B**), and 39 genes that combined (**C**) were subjected to PPI network analysis. In the bacterial growth assay, 24 genes whose expression levels were up-regulated by more than two-fold (**D**), 14 genes whose expression levels were down-regulated by more than two-fold (**E**), and 38 genes (**F**) that combined were subjected to PPI network analysis. Each circle represents a predicted protein, and the line represents the network.

We performed protein–protein interaction (PPI) network analysis to comprehensively predict interactions between protein molecules inferred from genes obtained by RNA sequencing analysis. PPI network analysis was performed on the 27 up-regulated genes and 12 down-regulated genes in the bacterial survival assay and the 24 up-regulated genes and 14 down-regulated genes in the growth assay. In the bacterial survival assay, only *ComYD* and *SMU_1980c* formed a network among the up-regulated genes, and no network was formed among the down-regulated genes (Figure 5A,B). For both up-regulated and down-regulated genes, networks were found between *ComYD* and *SMU_1980c*, and between *SMU_1141c* and *SMU_1671c* (Figure 5C). In the bacterial growth assay, only *SMU_382c* and *SMU_383c* formed a network among the up-regulated genes; no network was observed among the down-regulated genes, and only *SMU_382c* and *SMU_383c* formed a network among the up-and down-regulated genes (Figure 5D–F). Next, we used UniProt (2025_03) to analyze the functions of putative proteins synthesized from genes identified as interacting in the PPI network analysis (Table 5). The protein synthesized from *comYD* was found to be involved in the establishment of competence for transformation by responding to stimuli as a biological process. Both proteins synthesized from *SMU_382c* and *SMU_383c* were found to be involved in catalytic activity, with *SMU_382c* exhibiting oxidoreductase activity and *SMU_383c* exhibiting aldehyde dehydrogenase activity.

### 2.6. Gene Ontology (GO) Enrichment Analysis with Genes Whose Expression Was Altered by AITC Revealed Functional Interpretations

**Figure 6:**
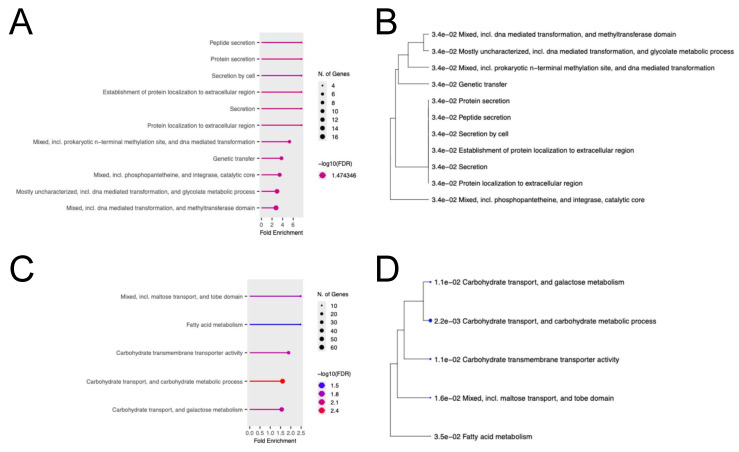
Gene Ontology (GO) enrichment analysis using genes whose expression was altered in *S. mutans* in the presence of at least one condition of 0.001%, 0.01%, or 0.1% AITC compared to the absence of AITC. In the bacterial survival assay, 278 genes whose expression levels were up- or down-regulated by more than 2.5-fold. Chart (**A**) and a hierarchical clustering tree summarizing correlations between pathways (**B**). In the bacterial growth assay, 819 genes whose expression levels were up- or down-regulated by more than 2.5-fold. Chart (**C**) and a hierarchical clustering tree summarizing correlations between pathways (**D**). Larger dots indicate more significant *p* values.

GO enrichment analysis was performed using genes whose expression was altered in *S. mutans* in the presence of at least one condition of 0.001%, 0.01%, or 0.1% AITC compared to the absence of AITC. In the bacterial survival assay, 278 genes whose expression levels were up- or down-regulated by more than 2.5-fold were identified as being related to protein or peptide secretion and biochemical pathways, including DNA-mediated transformation (Figure 6A,B). In the bacterial growth assay, 819 genes whose expression levels were up- or down-regulated by more than 2.5-fold were found to be related to biochemical pathways, including carbohydrate transport and metabolism (Figure 6C,D).

## 3. Discussion

AITC, isolated initially from cruciferous plants, has been applied in the medical and food industries for its excellent anticancer and antimicrobial activities [20]. AITC also has bactericidal activities against human pathogenic bacteria such as *Salmonella Montevideo*, *Escherichia coli* O157:H7, and *Listeria monocytogenes* Scott A [21]. In the present study, we demonstrated that AITC has inhibited cariogenicity in all respects of survival, growth, and biofilm formation on *S. mutans*, a major commensal and caries-associated bacterium in humans. We also clarified the effect of AITC on gene expression in *S. mutans* by applying bioinformatics analyses.

### 3.1. Inhibitory Effect

In the bacterial survival assay without a nutrient environment, AITC showed a significant anti-survival effect at a concentration of 0.1% for 3 h, and the bacterial count was decreased in all concentrations after 24 h of incubation. In addition, in a bacterial growth assay with a nutrient-rich environment, the growth of *S. mutans* was inhibited in an AITC concentration-dependent manner. In addition, the amount of biofilm was also significantly decreased with 0.1% AITC. These in vitro inhibitory effects of AITC were observed after several hours. Another antibacterial substance that showed effects after several hours in vitro was also found to reduce *S. mutans* in saliva in a human in vivo study when used as a regular mouthwash [22]. Therefore, AITC may effectively inhibit *S. mutans* in the human oral cavity, although further clinical investigation is required to apply AITC in dental caries prevention products.

### 3.2. Concentration

AITC at tens of μM is cytotoxic to tumor cells and is expected to be used as an anticancer substance [23,24]. The molecular weight of AITC is 99.15, and a concentration of tens of μM corresponds to a concentration slightly lower than the 0.001% used in our study. These studies also highlight the need for additional research using normal cells. Still, these studies are conducted on the assumption that low concentrations of AITC are not toxic to healthy tissues composed of normal cells. In animal experiments, mice were given free access to drinking water containing tens to hundreds of μM AITC [25]. Thus, transient administration of AITC at the concentration used in this study is expected to be highly safe clinically.

Low concentrations of antibiotics can promote biofilm formation [26,27]. We performed a biofilm assay using *S. mutans* with the addition of 0.001%, 0.01%, and 0.1% AITC. As a result, 0.1% AITC significantly inhibited biofilm formation, and no significant difference was observed between the other AITC concentrations and the control group. The minimum inhibitory concentration (MIC) of major gram-positive facultative anaerobic oral bacteria against AITC has been reported to be 0.1% to 0.4% [28]. Thus, it is unlikely that AITC promotes biofilm formation. However, multiple species of bacteria form actual biofilms, and future evaluations of the effect of AITC on biofilm formation using models that are closer to clinical conditions are necessary.

### 3.3. Analysis of Inhibitory Mechanism

RNA sequencing analysis showed that, except for 0.1% AITC in the bacterial growth assay, each concentration of AITC in the bacterial survival and growth assays induced a two-fold or greater change in the expression of approximately 150–200 *S. mutans* genes compared to the absence of AITC. *S. mutans* consists of approximately 2000 genes [29], and approximately 10% of the genes were affected by each concentration of AITC. Only 0.1% AITC in the growth assay induced changes in nearly 1000 gene expressions, approximately half of the total *S. mutans* genes. The 0.1% AITC drastically reduced the number of *S. mutans* in both the bacterial survival assay and the growth assay, significantly affecting the genes of *S. mutans*, especially during growth. Among these genes, those presumed to be functionally important should be identified, and their expression levels should be confirmed using quantitative real-time PCR (qRT-PCR). Furthermore, in vitro studies using *S. mutans* with those genes knocked out may provide valuable insights. In addition, in the oral cavity, *S. mutans* does not act alone, and several other microbes contribute to cariogenic biofilm formation [30]. Future research focuses on other microbes that form polymicrobial oral biofilms.

There were approximately 40 *S. mutans* genes whose expression was commonly altered by AITC at each concentration in both the bacterial survival and growth assays. We searched for these genes in PubMed and found that only *comYD*, a member of the ComY protein family involved in regulating genetic competence, had a known function [31]. Many streptococcal species can undergo natural transformation by entering a temporary physiological state known as genetic competence [32,33]. This genetic competence plays a vital role in *S. mutans*, which affects cell survival. Among the genes involved in competence, the *comY* operon, including *comYD*, is classified as a late competence gene and is activated by the early competence gene *comX* [34]. The expression of *comX* can vary depending on environmental factors [35,36,37], which may affect the expression of the *comY* operon. In this study, AITC did not affect the expression of *comX* but only altered the expression of *comYD* and *SMU_1980*, a hypothetical protein located immediately downstream of *comYD* and conserved in other *Streptococcus* species [34]. This result suggests that AITC acts on *comYD* and its neighboring genes, independent of *comX*, resulting in a decrease in the bacterial count of *S. mutans*.

We focused on these genes and clarified the predicted interactions of the proteins synthesized from them by PPI network analysis, following previous studies using other antibacterial substances [7,38]. As a result of the PPI network analysis, AITC detected *comYD* in bacterial survival assays, but the number of proteins that showed interactions in the PPI network analysis was minimal compared to previous studies [12,31], and there was almost no information regarding the proteins and their functions. Next, we used the UniProt database to search for the functions of proteins predicted to be synthesized from genes that had interactions in the PPI network analysis. As a result, the proteins synthesized from *SMU_382c* and *SMU_383c* had catalytic activity involving oxidoreductase and aldehyde dehydrogenase, respectively. Catalytic activity involving bacterial enzymes has a significant effect on bacterial growth by affecting nutrient utilization, metabolic pathways, and responses to environmental stress [39,40]. Therefore, AITC can reduce the growth ability by affecting catalytic activity through changes in the expression of *SMU_382c* and *SMU_383c*.

RNA sequencing analysis and UniProt analysis showed that some of the genes whose expression was altered by AITC were homologous to genes in *Streptococcus* species other than *S. mutans*. Therefore, AITC may also have an inhibitory effect on other *Streptococcus* species, the most dominant bacterial species in the oral cavity [41,42]. To clarify the extent of AITC’s effect on oral bacteria and the associated changes in the microbiome, human clinical studies are necessary. Furthermore, AITC may have a different inhibitory mechanism against *S. mutans* than existing antibacterial substances and may be effective against *Streptococcus* species that have acquired resistance to antibacterial substances. In addition, we must also pay close attention to the acquisition of bacterial resistance to AITC.

GO enrichment analysis is a method to investigate the biological significance of a group of genes [43]. In this study, we analyzed the biological functions of *S. mutans* affected by AITC based on the results of gene expression analysis of AITC-induced *S. mutans*. The bacterial survival assay demonstrated that AITC impacted protein or peptide secretion and biochemical pathways, including DNA-mediated transformation, which are crucial for bacterial survival [44,45]. In addition, the bacterial growth assay demonstrated that AITC influenced biochemical pathways, including carbohydrate transport and metabolism, which are essential for bacterial growth [46].

**Figure 7:**
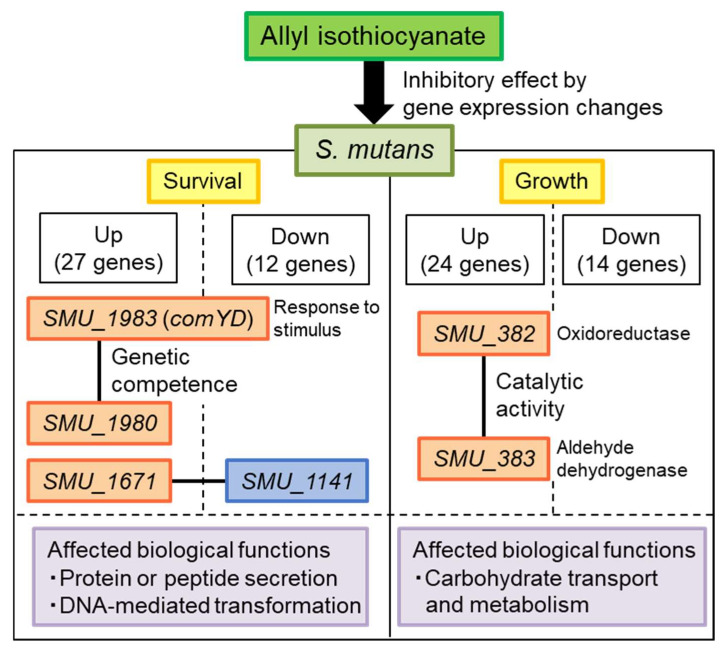
Schema of inhibitory effect of AITC on *S. mutans*.

Our results suggest that AITC induces noticeable expression changes in genes of unknown function in *S. mutans*, indicating that AITC inhibits *S. mutans* by a novel mechanism different from conventional antibacterial substances (Figure 7). AITC affected 39 genes (27 up-regulation and 12 down-regulation) in killing *S. mutans* and 38 genes (24 up-regulation and 14 down-regulation) in growth inhibition. Among these genes, interactions were observed between *SMU_1983* (*comYD*) and *SMU_1980*, *SMU_1671* and *SMU_1141*, and *SMU_382* and *SMU_383*. *SMU_1983* (*comYD*) responds to stimuli and, together with *SMU_1980*, is involved in controlling genetic competence related to survival. *SMU_382c* and *SMU_383c* have catalytic activities related to oxidoreductase and aldehyde dehydrogenase, respectively, which are involved in bacterial growth. AITC’s effects on these interactions in *S. mutans* may result in a decrease in survival rate and growth inhibition of *S. mutans*. In addition, analysis of entire genes in S. mutans whose expression was altered by AITC revealed that AITC affected biological functions essential for survival and growth, including protein or peptide secretion, DNA-mediated transformation, carbohydrate transport, and metabolism.

### 3.4. Comparison with Existing Drugs

It has been reported that AITC has lower antibacterial activity against oral pathogens compared to chlorhexidine [28]. However, natural ingredients can enhance their physical properties and improve their antibacterial effects by modifying their structure or combining them with other substances [11,47]. Therefore, future improvements in AITC are expected. While many conventional antibacterial drugs, including chlorhexidine, are highly effective, there are also reports of potential side effects [48,49]. Currently, AITC is more expensive than chlorhexidine; however, costs are expected to be reduced through mass production as clinical applications progress. In addition, many natural substances derived from plants are considered safer than conventional synthetic antibacterial drugs and may be effective in controlling bacteria that are resistant to existing drugs [49,50]. Although AITC is currently inferior to chlorhexidine in terms of effectiveness and cost, it is likely to have a higher safety profile, and future research and applications are expected to progress.

### 3.5. Current Limitations and Future Challenges for Clinical Application

The clinical application of AITC is hindered by its volatility, instability, low solubility, low viability, and odor, which makes the delivery of AITC a challenging task [16,51]. Sato et al. (2009) investigated the isothiocyanates produced by red turnips stored under various conditions [52]. They reported that when stored at room temperature for one year, the number of components decreased by 70% even when protected from light and by 90% when not protected from light. The lowest rate of decline was observed in refrigerated, light-protected conditions, suggesting that storage conditions also have a significant impact on AITC. In our study, AITC was stored at 4 °C, and the solution was adjusted for each experiment and used immediately. Additionally, all experiments were carried out in a sealed environment.

Recent research has been conducted on producing AITC in situ using the precursor of AITC, sinigrin, as a prodrug [47]. Minato et al. (2024) reported that injection of AITC into the gingiva of mice with induced periodontitis inhibited alveolar bone resorption [53]. The clinical effectiveness of AITC has been demonstrated; however, challenges remain, including temperature control and stability independent of solvents. Furthermore, research on AITC spans the fields of microbiology, chemistry, and public health; therefore, collaboration with various scientists and specialists is essential to apply AITC in clinical practice.

## 4. Materials and Methods

### 4.1. S. mutans Strain and Culture Conditions

The *S. mutans* strain MT8148 (serotype c) was cultured on Mitis salivarius agar (Difco Laboratories, Detroit, MI, USA) plates containing bacitracin (0.2 U/mL; Sigma-Aldrich, St. Louis, MO, USA) and 15% (*w*/*v*) sucrose (MSB agar) [11,12]. The strain was inoculated into BHI broth (Difco Laboratories) and cultured at 37 °C for 18 h for use in this study.

### 4.2. Materials

AITC was purchased from Tokyo Chemical Industry Co., Ltd. (Tokyo, Japan) and diluted to 5 mM/mL with dimethyl sulfoxide (DMSO). The dilutions were further adjusted to final AITC concentrations of 0.001%, 0.01%, and 0.1% for the experiments. The negative control was 0% AITC, and the same amount of DMSO was added as in the other conditions.

### 4.3. Bacterial Survival Assay

A bacterial survival assay was performed using the previously described methods with some modifications [11]. Cultured bacteria were collected by centrifugation at 7000× *g* at 4 °C for 10 min. The cultures were washed and resuspended in PBS to reach 1 × 10^9^ colony-forming units (CFUs)/mL with 0%, 0.001%, 0.01%, and 0.1% of AITC. Bacterial suspensions were incubated at 37 °C for 5 min, 30 min, 3 h, 6 h, 12 h, and 24 h on static. These bacterial suspensions were spread onto MSB agar plates using the spread plate technique and anaerobically cultured at 37 °C for 48 h. After this incubation period, the number of colonies was counted, and the bacterial count was calculated according to the dilution rate.

### 4.4. Bacterial Growth Assay

Bacterial growth assays were performed using previously described methods, with some modifications [11]. Cultured bacteria were added to BHI broth at a final concentration of 1.0 × 10^7^ CFUs/mL. Bacterial suspensions were cultured with 0%, 0.001%, 0.01%, and 0.1% AITC at 37 °C, and the OD_550_ values were measured using an absorptiometer. The turbidity change was determined as follows: {(turbidity with bacterial suspensions at each timepoint) − (initial turbidity without bacterial suspensions)}.

In addition, the bacterial suspensions at 24 h were spread onto MSB agar plates using the spread plate technique and anaerobically cultured at 37 °C for 48 h; the numbers of colonies were then counted. The bacterial growth rate was calculated by counting the number of bacterial colonies as follows: {(number of bacterial colonies with AITC)/(number of bacterial colonies without AITC) × 100}.

### 4.5. Biofilm Assay

Biofilm assays were performed using a previously described method, with some modifications [11]. Cultured bacteria were added to BHI broth containing 1% sucrose at a final concentration of 1.0 × 10^6^ CFUs/mL. Bacterial suspensions were incubated at 37 °C for 24 h with 0%, 0.001%, 0.01%, 0.1% of AITC. The tube was washed with PBS to remove unbound bacteria. Biofilms were stained with crystal violet for 1 min at room temperature. After washing with PBS, the biofilm was dissolved in 95% (*v*/*v*) ethanol. Then, the biofilm was quantified by measuring the OD_595_ values with an absorptiometer.

### 4.6. RNA Sequencing Analysis

*S. mutans* were treated with AITC at concentrations of 0%, 0.001%, 0.01%, or 0.1% for 24 h. Bacterial cells were lysed using Qiazol (Qiagen, Germantown, MD, USA), and the total RNA of *S. mutans* was isolated using miRNeasy Micro Kit (Qiagen, Germantown, MD, USA) according to the manufacturer’s instructions. Library preparation was performed using a GenNext RamDA-seq Single Cell Kit (Toyobo, Tokyo, Japan). Whole transcriptome sequencing was executed with the Illumina NovaSeq 6000 platform (Illumina Inc., San Diego, CA, USA) in a 100-base single-end mode. Sequenced reads were mapped to the reference genome sequences (*S. mutans* UA159) using HISAT2 ver. 2.1.0. Counts per gene were calculated with featureCounts v2.0.0.

### 4.7. Bioinformatics Analyses

The StringApp12.0 (Search Tool for the Retrieval of Interacting Genes/Proteins) online database (https://string-db.org/) was accessed on 1 April 2025. Protein–protein interaction (PPI) networks were constructed and visualized using 39 genes in the survival assay (27 up-regulated and 12 down-regulated) and 38 genes in the growth assay (24 up-regulated and 14 down-regulated) that showed more than 2-fold expression changes in all three conditions (0.001%, 0.01%, 0.1%) with AITC compared to those without AITC in RNA sequencing. In addition, UniProt (https://www.uniprot.org) was accessed on 20 June 2025. Genes that were found to interact in the PPI network analysis were entered into UniProt to investigate the functions of the proteins predicted to be synthesized from these genes. GO enrichment analysis was performed using ShinyGO 0.82 online resources (https://bioinformatics.sdstate.edu/go/) accessed on 27 June 2025, as described previously [12]. A *p* value cutoff of 0.05 for the false discovery rate was used to determine the genes used for GO enrichment analysis.

### 4.8. Statistical Analysis

Statistical analyses were conducted using GraphPad Prism 9 (GraphPad Software Inc., La Jolla, CA, USA). Results were presented as means ± standard error. Statistical significance was determined using the Kruskal–Wallis test for nonparametric analysis, followed by the Dunn test for multiple comparisons, and was significantly different at *p* < 0.05.

## 5. Conclusions

AITC had a high inhibitory effect on the survival, growth, and biofilm formation of *S. mutans*. RNA sequencing and bioinformatics analyses showed that AITC mainly acted on *S. mutans* genes of unknown functions, though some of the genes were involved in inhibiting the survival and growth of *S. mutans*. Our results suggest that AITC inhibits *S. mutans* through its unique inhibitory mechanism and may be used as a new substance for preventing dental caries. Although there are various issues with AITC, including its volatility or odor, further research may develop clinical application of AITC in the dental field in the future.

## Figures and Tables

**Table 1:** List of up-regulated genes of *S. mutans* at 0.001%, 0.01%, and 0.1% AITC compared to the absence of AITC in the bacterial survival assay using RNA sequencing analysis. Fold changes were calculated by signal in each concentration of AITC/signal without AITC. Bold in the gene name column indicates that the fold change was more than 100 at each concentration of AITC. Bold in the fold change column indicates a fold change of more than 100.

		Fold Change		
Gene Name	Product	AITC 0.001%	AITC 0.01%	AITC 0.1%
*SMU_1341c*	putative gramicidin S synthetase	3.128	3.517	2.162
*SMU_677*	putative transcriptional regulator (MerR family)	5.758	4.382	2.307
*SMU_790*	hypothetical protein	2.879	2.191	2.401
*SMU_1942c*	putative amino acid binding protein	2.720	2.009	2.798
*SMU_1065c*	putative transcriptional regulator (GntR family)	2.506	3.104	3.335
*SMU_984*	hypothetical protein	2.112	2.739	3.481
*SMU_1141c*	hypothetical protein	2.150	4.659	3.484
*SMU_386*	putative ribosomal-protein-alanine acetyltransferase	4.072	2.858	3.868
*SMU_1621c*	hypothetical protein	3.272	3.706	4.242
*SMU_2115*	putative short-chain dehydrogenase	4.799	6.035	4.802
*SMU_174c*	hypothetical protein	2.160	2.189	5.405
*SMU_1774c*	hypothetical protein	3.841	4.385	7.207
*SMU_1780*	hypothetical protein	6.727	7.679	9.617
*SMU_890*	hypothetical protein	8.656	7.685	12.031
*SMU_1983* (*comYD*)	putative competence protein ComYD	5.710	3.037	12.204
*SMU_t36*	tRNA-Ser	2.399	4.382	12.604
*SMU_1980c*	hypothetical protein	27.975	3.112	17.253
*SMU_2134*	putative transcriptional regulator	33.375	29.623	18.550
*SMU_976* (*potD*)	putative ABC transporter, periplasmic spermidine/putrescine-binding protein	37.522	57.046	24.312
*SMU_1552c*	hypothetical protein	3.626	27.443	30.070
*SMU_1643c*	hypothetical protein	22.405	42.631	37.369
*SMU_136c*	putative transcriptional regulator	25.906	9.852	64.797
*SMU_1087*	putative 4-oxalocrotonate tautomerase	**349.836**	**399.330**	70.345
***SMU_1353***	putative transposase	**759.405**	**247.650**	**135.672**
***SMU_1152c***	hypothetical protein	**141.089**	**140.197**	**169.344**
***SMU_t34***	tRNA-Ser	**3937.040**	**6291.730**	**7878.940**
***SMU_t38***	tRNA-Gln	**13** **,594.000**	**12** **,069.100**	**18** **,892.200**

**Table 2:** List of down-regulated genes of *S. mutans* at 0.001%, 0.01%, and 0.1% AITC compared to the absence of AITC in the bacterial survival assay using RNA sequencing analysis. Fold changes were calculated by signal in each concentration of AITC/signal without AITC. Bold in the gene name column indicates that the fold change was more than −100 at each concentration of AITC. Bold in the fold change column indicates a fold change of more than −100.

		Fold Change		
Gene Name	Product	AITC 0.001%	AITC 0.01%	AITC 0.01%
*SMU_t64*	tRNA-Glu	−2.084	**−3695.480**	**−3695.480**
*SMU_738*	hypothetical protein	−2.085	**−361.099**	**−361.099**
***SMU_1369***	hypothetical protein	**−267.274**	**−267.274**	**−267.274**
***SMU_2073c***	hypothetical protein	**−206.566**	**−206.566**	**−206.566**
*SMU_1516* (*covS*)	putative histidine kinase CovS; VicK-like protein	−3.413	**−159.146**	**−159.146**
*SMU_1360c*	hypothetical protein	**−105.478**	−2.327	**−105.478**
*SMU_1500* (*rexB*)	putative exonuclease RexB	−4.356	**−111.655**	−3.110
*SMU_05*	hypothetical protein	−9.701	**−489.950**	−2.518
*SMU_1671c*	hypothetical protein	−2.747	−5.295	−2.416
*SMU_982* (*bglB2*)	putative BglB fragment	−2.214	−3.104	−2.361
*SMU_412c*	putative Hit-like protein involved in cell-cycle regulation	−5.706	−2.199	−2.330
*SMU_1294* (*flaW*)	putative flavodoxin	−3.460	−56.287	−2.075

**Table 3:** List of up-regulated genes of *S. mutans* at 0.001%, 0.01%, and 0.1% AITC compared to the absence of AITC in the bacterial growth assay using RNA sequencing analysis. Fold changes were calculated by signal in each concentration of AITC/signal without AITC. Bold in the gene name column indicates that the fold change was more than 100 at each concentration of AITC. Bold in the fold change column indicates a fold change of more than 100.

		Fold Change		
Gene Name	Product	AITC 0.001%	AITC 0.01%	AITC 0.1%
*SMU_975* (*potC*)	putative spermidine/putrescine ABC transporter, permease protein	2.542	2.850	2.155
*SMU_40*	conserved hypothetical protein	4.581	5.199	2.534
*SMU_1694c*	putative permease	2.231	2.120	2.569
*SMU_10*	conserved hypothetical protein	3.192	2.741	3.359
*SMU_1504c*	hypothetical protein	3.088	3.004	3.578
*SMU_677*	putative transcriptional regulator (MerR family)	4.617	2.414	3.985
*SMU_605*	hypothetical protein	7.036	6.022	4.183
*SMU_925*	hypothetical protein	2.307	2.156	6.673
*SMU_219*	hypothetical protein	4.394	3.506	8.167
*SMU_2153c*	putative peptidase	2.143	2.003	11.997
*SMU_1622* (*pmsR*)	putative peptide methionine sulfoxide reductase	3.725	3.278	13.382
*SMU_1355c*	putative transposase fregment	2.649	3.007	17.162
*SMU_1287*	putative transcriptional regulator	2.649	4.010	18.061
*SMU_407*	conserved hypothetical protein	3.175	3.986	19.023
*SMU_382c*	putative oxidoreductase	2.037	5.078	24.988
*SMU_383c*	conserved hypothetical protein; putative reductase	2.171	4.262	25.245
*SMU_1201c*	conserved hypothetical protein	87.466	95.263	85.115
*SMU_136c*	putative transcriptional regulator	15.933	9.040	87.519
*SMU_1526c*	conserved hypothetical protein	**106.669**	40.362	**132.068**
***SMU_1024c***	putative transposase fragment	**174.941**	**198.571**	**133.015**
*SMU_656* (*mutE2*)	putative MutE	33.486	33.501	**151.823**
*SMU_379*	hypothetical protein	88.063	**176.696**	**236.467**
***SMU_1353***	putative transposase	**199.926**	**113.588**	**258.676**
***SMU_t35***	tRNA-Leu	**2832.920**	**1071.800**	**7180.290**

**Table 4:** List of down-regulated genes of *S. mutans* at 0.001%, 0.01%, and 0.1% AITC compared to the absence of AITC in the bacterial growth assay using RNA sequencing analysis. Fold changes were calculated by signal in each concentration of AITC/signal without AITC. Bold in the gene name column indicates that the fold change was more than −100 at each concentration of AITC. Bold in the fold change column indicates a fold change of more than −100.

		Fold Change		
Gene Name	Product	AITC 0.001%	AITC 0.01%	AITC 0.1%
***SMU_t25***	tRNA-Glu	**−1855.060**	**−1855.060**	**−1855.060**
***SMU_t38***	tRNA-Gln	**−1580.070**	**−1580.070**	**−1580.070**
***SMU_t33***	tRNA-Leu	**−1023.210**	**−1023.210**	**−1023.210**
***SMU_1231c***	hypothetical protein	**−672.496**	**−672.496**	**−672.496**
***SMU_1310***	hypothetical protein	**−176.481**	**−176.481**	**−176.481**
*SMU_1554c*	hypothetical protein	−98.370	−98.370	−98.370
*SMU_711*	conserved hypothetical protein	−2.645	−3.496	−51.212
*SMU_1714c*	hypothetical protein	−27.984	−27.984	−27.984
*SMU_1508c*	putative coenzyme PQQ synthesis protein	−2.455	−22.668	−22.668
*SMU_959c*	hypothetical protein	−3.507	−6.431	−6.998
*SMU_189*	hypothetical protein	−42.282	−42.282	−6.815
*SMU_277*	hypothetical protein	−3.401	−2.248	−6.710
*SMU_1087*	putative 4-oxalocrotonate tautomerase	−3.401	−5.994	−5.751
*SMU_2094c*	conserved hypothetical protein	−2.721	−5.996	−3.372

**Table 5:** Functional analysis of putative proteins synthesized from genes identified as interacting in PPI network analysis using UniProt.

		Annotations	
Assay	Gene Name	Molecular Function (Term)	Biological Process (Term)
Survival	*SMU_1980c*	-	-
Survival	*SMU_1983* (*comYD*)	-	Response to stimulus (Establishment of competence for transformation)
Survival	*SMU_1141c*	-	-
Survival	*SMU_1671c*	-	-
Growth	*SMU_382c*	Catalytic activity (Oxidoreductase activity)	-
Growth	*SMU_383c*	Catalytic activity (Aldehyde dehydrogenase activity)	-

## Data Availability

The raw data supporting the conclusions of this article will be made available by the authors on request.
